# Does improved nurse staffing impact patient outcomes in cancer? Association between chronic diseases and mortality among older adult patients with lung cancer in Korea

**DOI:** 10.1371/journal.pone.0301010

**Published:** 2024-05-08

**Authors:** Kyu-Tae Han, Seungju Kim

**Affiliations:** 1 Division of Cancer Control & Policy, National Cancer Control Institute, National Cancer Center, Goyang, Republic of Korea; 2 Department of Health System, College of Nursing, The Catholic University of Korea, Seoul, Republic of Korea; King Saud University Medical City, SAUDI ARABIA

## Abstract

**Background:**

Evidence regarding the impact of nurse staffing on the health outcomes of older adult patients with cancer is scarce. Therefore, this study aimed to evaluate the impact of nurse staffing on long-term and short-term mortality in elderly lung cancer patients.

**Methods:**

This study analyzed data from 5,832 patients with lung cancer in Korea from 2008 to 2018. Nursing grade was considered to assess the effect of nursing staff on mortality in older adult patients with lung cancer. The Cox proportional hazards model was used to evaluate the effect of the initial treatment hospital’s nursing grade on one- and five-year mortality. Additionally, economic status and treatment type of patients were analyzed.

**Results:**

Approximately 31% of older adult patients with lung cancer died within one year post-diagnosis. Patients in hospitals with superior nursing grades (lower nurse-to-bed ratios) exhibited lower mortality rates. Hospitals with nursing grades 2 and 3 exhibited approximately 1.242–1.289 times higher mortality than grade 1 hospitals. Further, the lower the nursing grade (higher nurse-to-bed ratio), the higher the five-year mortality rate.

**Conclusion:**

Both short- and long-term mortality rates for older adult patients with lung cancer increased at inferior nursing grades. Treatment in hospitals having inferior nursing grades, upon initial hospitalization, may yield better outcomes. This study provides valuable insight into the quality of adequate staffing to improve the quality of care for elderly cancer patients.

## Introduction

Cancer is an aging-related disease, and the number of cancer patients is increasing with the aging of the population [1]. In 2018, 2.3 million adults aged 80 years or older developed cancer, and, by 2050, 6.9 million people are expected to have cancer [2]. As the number of older adult cancer patients increases, appropriate management is important, and a multidisciplinary approach and continuity of care are required throughout the entire treatment process. Accordingly, the report from the United States Institute of Medicine’s Committee on Improving the Quality of Cancer Care has detailed the qualitative aspects of cancer treatment for older adults offering numerous recommendations (e.g., evidence-based treatment) and highlighting the importance of patient-centered care [3–5]. The importance of medical staff’s role in the overall treatment process of older adult patients with cancer has been emphasized, and researchers have noted the need to secure an appropriate level of well-trained medical staff [6].

Older adult cancer patients experience various changes due to disease and aging; they become vulnerable to falls due to reduced physical activity, and changes in the surrounding environment, such as unfamiliar hospitals or the absence of people close to them, can have a negative impact on their health [7]. Therefore, providing high-quality care to older adult patients with cancer in the early stages of hospitalization is important; this implies that sufficient nursing staff (as nurses are a key human resource in patient care) must be secured. Nurse shortages may lead to improper treatment, which can negatively impact patient outcomes [8, 9]. Previous research has also shown that for each increase in the average nurse workload per patient, the risk of mortality within 30 days of hospitalization increases by 7% [10]. This indicates that nurse staffing level is a major factor influencing patient outcomes.

The positive impact of nurse staffing on patient outcomes has been shown in a variety of studies [11, 12], with higher nurse staffing levels associated with reduced mortality [13, 14] and a shorter length of stay [15]. Further, a study evaluating the dose–response relationship between the patient-to-nurse ratio and postoperative complications indicated a reduction in complications when the patient-to-nurse ratio per shift was 5.4 [16]. Some states in the United States and Australia have legislated minimum nurse-to-patient ratios based on shifts and wards, and the introduction of these laws has proven that retaining nurses not only saves money, but also leads to positive patient outcomes [17–19]. These findings have provided evidence for nurse staffing’s impact on patient outcomes—suggesting that adequate nurse staffing levels are important for maintaining the quality of care—but evidence for its specific impact on older adult patients with cancer is scarce.

Since older adult patients with cancer often have physical vulnerabilities and comorbidities [20, 21], appropriate patient-to-nurse ratios during the hospitalization of chronically ill elderly cancer patients are important as it can lead to better patient outcomes. Lung cancer, in particular, has a higher incidence and mortality rate than other types of cancer in older adults [22, 23]; therefore, appropriate management is required upon hospitalization, and appropriate nursing staff is essential for patient education as well as relevant information vis-à-vis goal-setting [24], at the time of their initial hospitalization. Therefore, this study aimed to evaluate nurse staffing’s effect on the long- and short-term mortality of older adult patients with lung cancer in Korea and determine how to secure appropriate nurse staffing. A subgroup analysis was performed to evaluate the differences in nurse staffing’s effect on mortality, per patient characteristics.

## Methods

### Study population

The data used in this study were obtained from the National Health Insurance Services (NHIS) Senior Sampled Cohort for the year 2008 in Korea. The participants comprised randomly sampled individuals aged 60–80 years. Additionally, individuals who turned 60 years old throughout the 2009–2019 period were also added to the study. The total sample comprised 545,831 individuals; their data (including sex, age, and insurance premium) were collected from 2002 to 2019.

**Fig 1:**
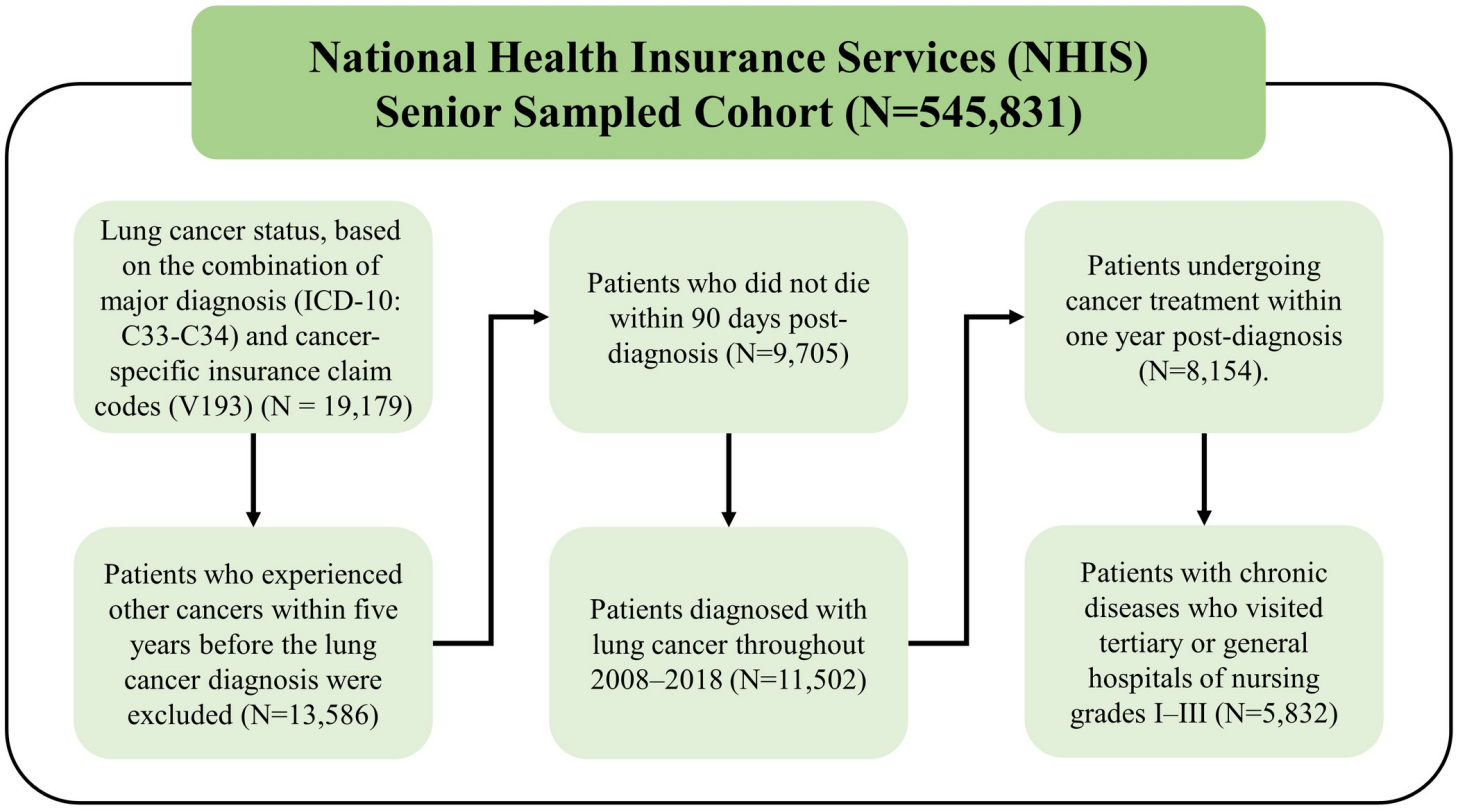
Selection of study population.

In this study, we included older adult lung cancer patients with chronic diseases and analyzed the relationship between mortality and nurse staffing. Lung cancer status was defined based on the combination of the major diagnosis (International Classification of Diseases [ICD]-10: C33–C34) and cancer-specific insurance claim codes (V193; N = 19,179). Exclusion criteria were patients diagnosed with carcinomas other than lung cancer, patients who died early, and patients who did not receive treatment. First, patients who had experienced other types of cancer within five years before their lung cancer diagnosis (N = 5,593) were excluded (N = 13,586) and only patients diagnosed with lung cancer from 2008–2018 were included (N = 11,502). Next, to avoid immortal time bias, only patients who did not die within 90 days post-diagnosis were included (N = 9,705). Further, only patients with lung cancer and a cancer treatment history (e.g., surgery, chemotherapy, and radiation therapy) at general hospitals within one year post-diagnosis were included (N = 8,154). Next, patients without medical details about chronic diseases including hypertension (ICD-10: I10–I15), dyslipidemia (ICD-10: E78), or diabetes mellitus (ICD-10: E10–E14) within one year before lung cancer diagnosis were excluded. Finally, only patients who visited hospitals of nursing grades 1–3 were included to avoid variation between hospitals (N = 5,832; Fig 1).

### Variables

The outcome variable in this study was one-year mortality. We defined the first date of lung cancer diagnosis (C33–C34 & V193) and collected patients’ data. If patients died within one year post-diagnosis, irrespective of the cause of death, they were classified into the “deceased” group; otherwise, they were classified into the “survivor” group.

The main independent variable in this study was the level of nurse staffing of the hospital for initial cancer treatment. Hospitals’ nurse staffing level was measured based on nursing grade. Considering the rationale that higher staffing leads to higher quality care, hospitals in Korea are assigned a nursing grade based on their nurse-to-bed ratio (tertiary hospitals: grade 1 = 1: 2.0, grade 2 = 1:2.5, grade 3 = 1:3.0, grade 4 = 1:3.5, grade 5 = 1:4.0; general hospitals: grade 1 = 1:2.5, grade 2 = 1:3.0, grade 3 = 1:3.5, grade 4 = 1:4.0, grade 5 = 1:4.5) [25]. Hospitals with a higher number of nurses per beds can be reimbursed additional medical costs under the National Health Insurance (NHI) system. In this study, we only included patients who were treated at hospitals with nursing grades of 1–3; notably, most tertiary hospitals providing cancer care in Korea have a nursing grade of 3 or above. The nursing grade of the hospital where patients with lung cancer received initial cancer treatment (including surgery, chemotherapy, or radiation therapy) was determined for each patient.

The other independent variables used in this study included the type, location, and size, of hospitals as well as the sex, age, type of insurance coverage, economic status, residence area, Charlson Comorbidity Index (CCI) score, year of diagnosis, and type of cancer treatment of patients. The hospitals that provided first cancer treatment were classified by type (tertiary or general), location (capital, metropolitan, or rural), and size (number of beds; based on 33rd percentiles [690 beds] and 66th percentiles [1,786 beds]) to assess the quality of initial cancer care. In Korea, approximately 97% of people are covered by the NHI, while insurance coverage is divided into three types based on people’s economic status and employment (NHI employee or self-employed). First, the NHI employee category comprises employee, employer, and their household members; they pay an NHI insurance premium based on their income. Second, the NHI self-employed category comprises all other individuals who pay an insurance premium based on their income, property, and standard of living. Thus, insurance premiums reflect individual income. In this study, based on their insurance premiums, patients were divided into four levels to consider the variation of economic status. The 3% as rest of NHI among Koreans is defined as Medical-Aid. The Medical-Aid group includes low-income or disabled individuals who do not pay insurance premiums. In Korea, a cancer-specific insurance claim code (V193) is assigned to medical claim data; NHI beneficiaries—including NHI employees or NHI self-employed individuals—only pay a 5% co-payment for medical costs associated with cancer care, while the Medical-Aid group pays 0% of inpatient care and 0–5% of outpatient care costs; other types of care generally have a 20–30% co-payment rate under the NHI.

To determine the participants’ clinical severity, the Charlson Comorbidity Index (CCI) score was calculated in this study. When calculating the CCI, cancer and cardiovascular diseases (e.g., myocardial infarction, congestive heart failure, peripheral vascular disease, and cerebrovascular disease) were excluded based on the study population’s definition. Medical record data related to comorbidities were limited to 90 days after the date of the first lung cancer diagnosis. The type of treatment was determined by whether patients receiving surgical treatment also received additional treatment (e.g., chemotherapy or radiation therapy) one year post-diagnosis. This assessment aimed to evaluate each patient’s clinical severity at diagnosis; the medical claim data did not include information on cancer stage.

### Statistical analysis

To identify the sample’s distribution, first, we examined the frequencies and percentages of the independent variables and conducted chi-square tests. Second, to compare patients’ survival time, Kaplan-Meier survival curves with log-rank tests were used according to hospitals’ nursing grade. Third, survival analysis using Cox proportional hazard model was performed to analyze the association between one-year mortality and nursing grade, while adjusting for other covariates. Additionally, survival analysis for five-year mortality was performed to investigate long-term outcomes by nursing grade. Finally, to compare the differences in the association between one-year mortality and nursing grade, subgroup analyses were performed, considering economic status (based on insurance premium) and type of treatment. All statistical analyses in this study were performed using SAS statistical software version 9.4 (Cary, NC).

### Ethics approval and consent to participate

This study utilized secondary data, and all patients’ personal data were encrypted and anonymized. This study was approved by the Catholic University Institutional Review Board. All methods were carried out in accordance with relevant guidelines and regulations.

## Results

**Fig 2:**
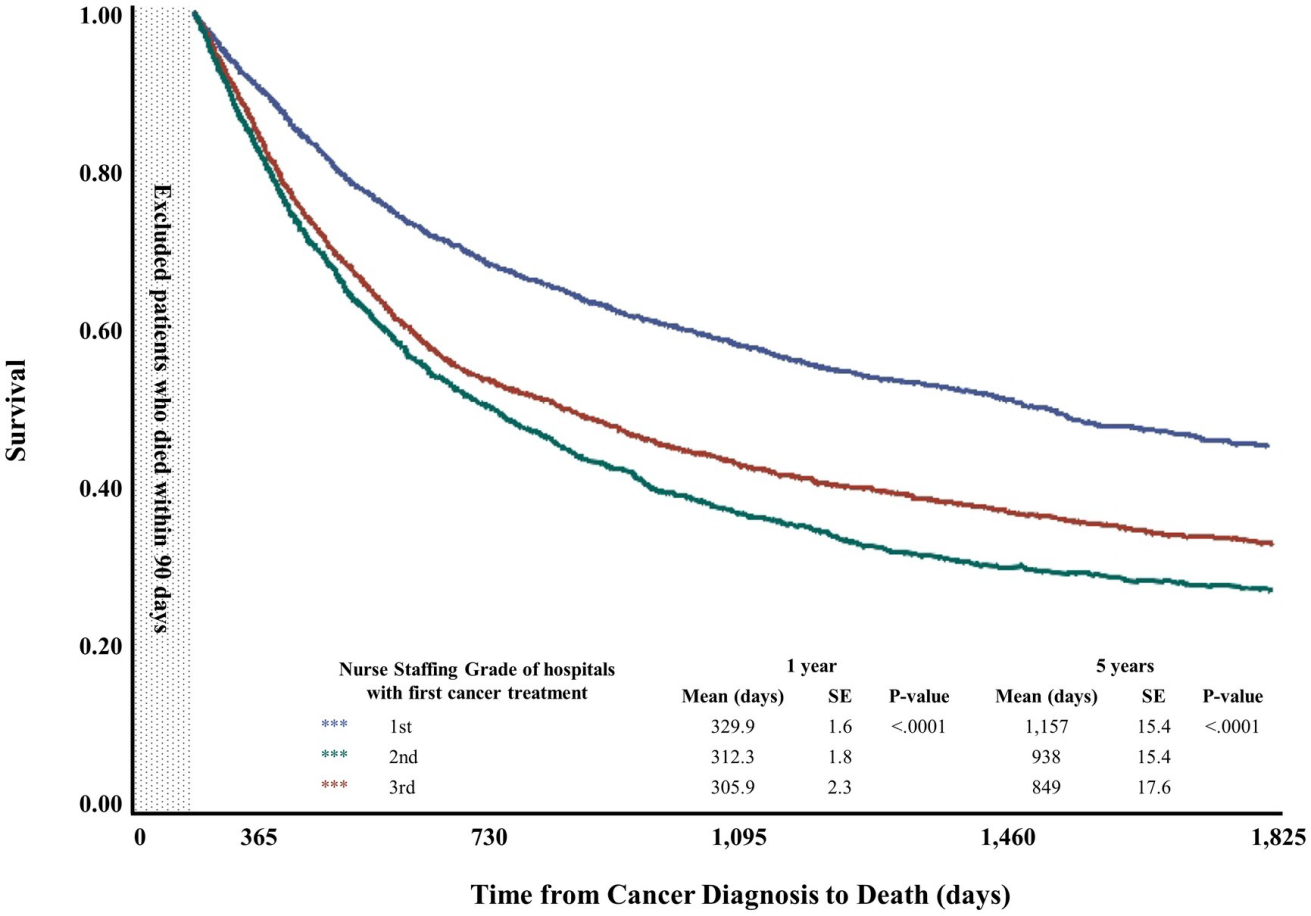
Kaplan-Meier survival curves by initial-treatment hospital’s nursing grade.

The sample comprised 5,832 patients having lung cancer with chronic diseases who visited hospitals for cancer treatment. Table 1 presents the study population’s distribution by one-year mortality. Approximately 30.9% (n = 1,801) of patients died within one year after the first diagnosis of lung cancer. For the independent variables, patients treated at superior nursing-grade hospitals exhibited lower mortality rates than those treated at lower nursing-grade hospitals (grade 1: 22.6%, grade 2: 34.5%, grade 3: 37.4%; P-value < .0001). Patients cared for by tertiary hospitals or those in capital cities exhibited lower one-year mortality (tertiary: 28.3%, general: 37.2%; P-value < .0001; capital: 26.8%, metropolitan: 36.4%, rural: 39.0%; P-value < .0001). Additionally, hospital size was inversely associated with mortality (P-value < .0001). Women and younger patients exhibited lower mortality (P-value < .0001). Patients who lived in metropolitan or rural areas exhibited higher mortality (P-value: 0.002). By clinical status, patients with higher CCI scores exhibited higher mortality (0: 23.8%, 1: 28.9%, 2: 34.3%, 3+: 33.6%; P-value < .0001). Patients who received only surgical treatment exhibited lower mortality rates than those who received chemotherapy or radiation therapy (surgery: 4.2%, surgery with chemotherapy or radiation therapy: 20.4%, chemotherapy or radiation therapy: 47.5%; P-value < .0001). Fig 2 presents the Kaplan-Meier Survival curves; patients treated at hospitals with superior nursing grades exhibited longer survival times than those treated at lower nursing-grade hospitals (one-year mortality, grade 1: 329.9, grade 2: 312.3, grade 3: 305.9; P-value < .0001).

**Table 1:** Results of one-year mortality per participants’ general characteristics.

Variables	One-year mortality					
	Total	Survivor		Deceased		P-value
		N	%	N	%	
**Hospitals’ nurse staffing grade**						
Grade 1	2,132	1,651	77.4	481	22.6	< .0001
Grade 2	2,191	1,436	65.5	755	34.5	
Grade 3	1,509	944	62.6	565	37.4	
**Hospital type**						
Tertiary	4,156	2,979	71.7	1,177	28.3	< .0001
General	1,676	1,052	62.8	624	37.2	
**Hospital location**						
Capital	3,594	2,630	73.2	964	26.8	< .0001
Metropolitan	1,397	888	63.6	509	36.4	
Rural	841	513	61.0	328	39.0	
**Hospital size (number of beds)**						
Small	1,330	850	63.9	480	36.1	< .0001
Mid	2,989	1,990	66.6	999	33.4	
High	1,507	1,185	78.6	322	21.4	
**Sex**						
Male	4,098	2,634	64.3	1,464	35.7	< .0001
Female	1,734	1,397	80.6	337	19.4	
**Age (years)**						
> 60	579	554	95.7	25	4.3	< .0001
61–65	1,097	826	75.3	271	24.7	
66–70	1,362	953	70.0	409	30.0	
71–75	1,388	897	64.6	491	35.4	
< 75	1,406	801	57.0	605	43.0	
**Type of insurance coverage**						
Medical-Aid	289	182	63.0	107	37.0	0.068
NHI, Self-employed	1,728	1,200	69.4	528	30.6	
NHI, Employee	3,815	2,649	69.4	1,166	30.6	
**Economic status**						
Low	1,573	1,081	68.7	492	31.3	0.1518
Mid-low	1,202	809	67.3	393	32.7	
Mid-high	2,034	1,407	69.2	627	30.8	
High	1,023	734	71.7	289	28.3	
**Residential area**						
Capital	2,322	1,645	70.8	677	29.2	0.002
Metropolitan	1,385	976	70.5	409	29.5	
Rural	2,125	1,410	66.4	715	33.6	
**Charlson Comorbidity Index (excluding cancer and cardiovascular diseases)**						
0	869	662	76.2	207	23.8	< .0001
1	1,811	1,288	71.1	523	28.9	
2	1,514	994	65.7	520	34.3	
3+	1,638	1,087	66.4	551	33.6	
**Year of diagnosis**						
2009	389	266	68.4	123	31.6	0.073
2010	398	278	69.8	120	30.2	
2011	532	365	68.6	167	31.4	
2012	490	334	68.2	156	31.8	
2013	529	349	66.0	180	34.0	
2014	618	420	68.0	198	32.0	
2015	665	449	67.5	216	32.5	
2016	676	470	69.5	206	30.5	
2017	779	582	74.7	197	25.3	
2018	756	518	68.5	238	31.5	
**Type of cancer treatment**						
Surgery	1,412	1,352	95.8	60	4.2	< .0001
Surgery with chemotherapy or radiation therapy	1,324	1,054	79.6	270	20.4	
Chemotherapy or radiation therapy	3,096	1,625	52.5	1,471	47.5	
**Total**	5,832	4,031	69.1	1,801	30.9	

Table 2 presents the results of survival analysis while adjusting for covariates. Patients treated in hospitals with inferior nursing grades (2–3) exhibited about 1.242–1.289 times higher mortality rates than those treated in grade-1 hospitals (grade 2, hazard ratio (HR): 1.242, 95% CI: 1.075–1.434, P-value: 0.0032; grade 3, HR: 1.289, 95% CI: 1.081–1.538, P-value: 0.0048). Patients who visited general hospitals exhibited higher mortality rates than those treated in tertiary hospitals (HR: 1.255, 95% CI: 1.099–1.434, P-value: 0.0008). However, the location and size of hospitals exhibited no statistically significant association with one-year mortality.

**Table 2:** Relationship between nursing grade and one-year mortality.

Variables	Mortality			
	One year			
	HR	LCL	UCL	P-value
**Hospital nurse staffing grade**				
Grade 1	1.000	-	-	
Grade 2	1.242	1.075	1.434	0.0032
Grade 3	1.289	1.081	1.538	0.0048
**Hospital type**				
Tertiary	1.000	-	-	
General	1.255	1.099	1.434	0.0008
**Hospital location**				
Capital	1.000	-	-	
Metropolitan	1.133	0.954	1.347	0.1555
Rural	1.113	0.928	1.334	0.2481
**Hospital size (number of beds)**				
Low	0.848	0.688	1.045	0.1223
Mid	0.958	0.810	1.134	0.6211
High	1.000	-	-	-
**Sex**				
Male	1.558	1.382	1.757	< .0001
Female	1.000	-	-	
**Age (years)**				
> 60	1.000	-	-	-
61–65	4.742	3.146	7.149	< .0001
66–70	5.569	3.714	8.350	< .0001
71–75	6.636	4.431	9.938	< .0001
< 75	8.028	5.363	12.018	< .0001
**Type of insurance coverage**				
Medical-Aid	1.034	0.831	1.288	0.7631
NHI, Self-employed	0.995	0.897	1.104	0.9220
NHI, Employee	1.000	-	-	
**Economic status**				
Low	1.056	0.904	1.234	0.4924
Mid-low	1.152	0.987	1.346	0.0737
Mid-high	1.061	0.921	1.221	0.4129
High	1.000	-	-	-
**Residential area**				
Capital	1.000	-	-	
Metropolitan	0.823	0.694	0.975	0.0244
Rural	0.954	0.831	1.096	0.5074
**Charlson Comorbidity Index (excluding cancer and cardiovascular diseases)**				
0	1.000	-	-	
1	1.096	0.932	1.289	0.2667
2	1.319	1.120	1.552	0.0009
3+	1.318	1.120	1.551	0.0009
**Year of diagnosis (continuous; yearly)**	0.986	0.969	1.003	0.1123
**Types of cancer treatment**				
Surgery	1.000	-	-	-
Surgery with chemotherapy or radiation therapy	4.802	3.628	6.357	< .0001
Chemotherapy or radiation therapy	11.531	8.893	14.952	< .0001

*Note*. HR = hazard ratio; LCL = lower control limit; UCL = upper control limit.

Regarding patients’ characteristics, men and older adult patients exhibited higher mortality. Regarding economic status, the type of insurance coverage or insurance premium as economic status was not associated with mortality. CCI scores > 2 were positively associated with higher mortality (2, HR: 1.319, 95% CI: 1.120–1.552, P-value: 0.0009; 3+, HR: 1.318, 95% CI: 1.120–1.551, P-value: 0.0009; ref: 0). By type of treatment, patients who underwent chemotherapy or radiation therapy exhibited higher mortality than patients who only had surgery (surgery with chemotherapy or radiation therapy, HR: 4.802, 95% CI: 3.62–6.357, P-value < .0001; chemotherapy or radiation therapy, HR: 11.531, 95% CI: 8.893–14.952, P-value < .0001; ref: surgery). Based on the results for five-year mortality, a comparison of long-term outcomes revealed that hospitals with inferior nursing grades were associated with higher mortality than grade 1 hospitals (grade 2, HR: 1.166, 95% CI: 1.053–1.291, P-value: 0.0031; grade 3, HR: 1.207, 95% CI: 1.063–1.371, P-value: 0.0036; Table 3).

**Table 3:** Relationship between nursing grade and five-year mortality.

Variables	Mortality			
	Five years			
	HR	LCL	UCL	P-value
**Hospital nurse staffing grade**				
Grade 1	1.000	-	-	-
Grade 2	1.166	1.053	1.291	0.0031
Grade 3	1.207	1.063	1.371	0.0036

†The results of survival analysis adjusting other covariates.

*Note*. Abbreviations: HR = hazard ratio; LCL = lower control limit; UCL = upper control limit.

**Fig 3:**
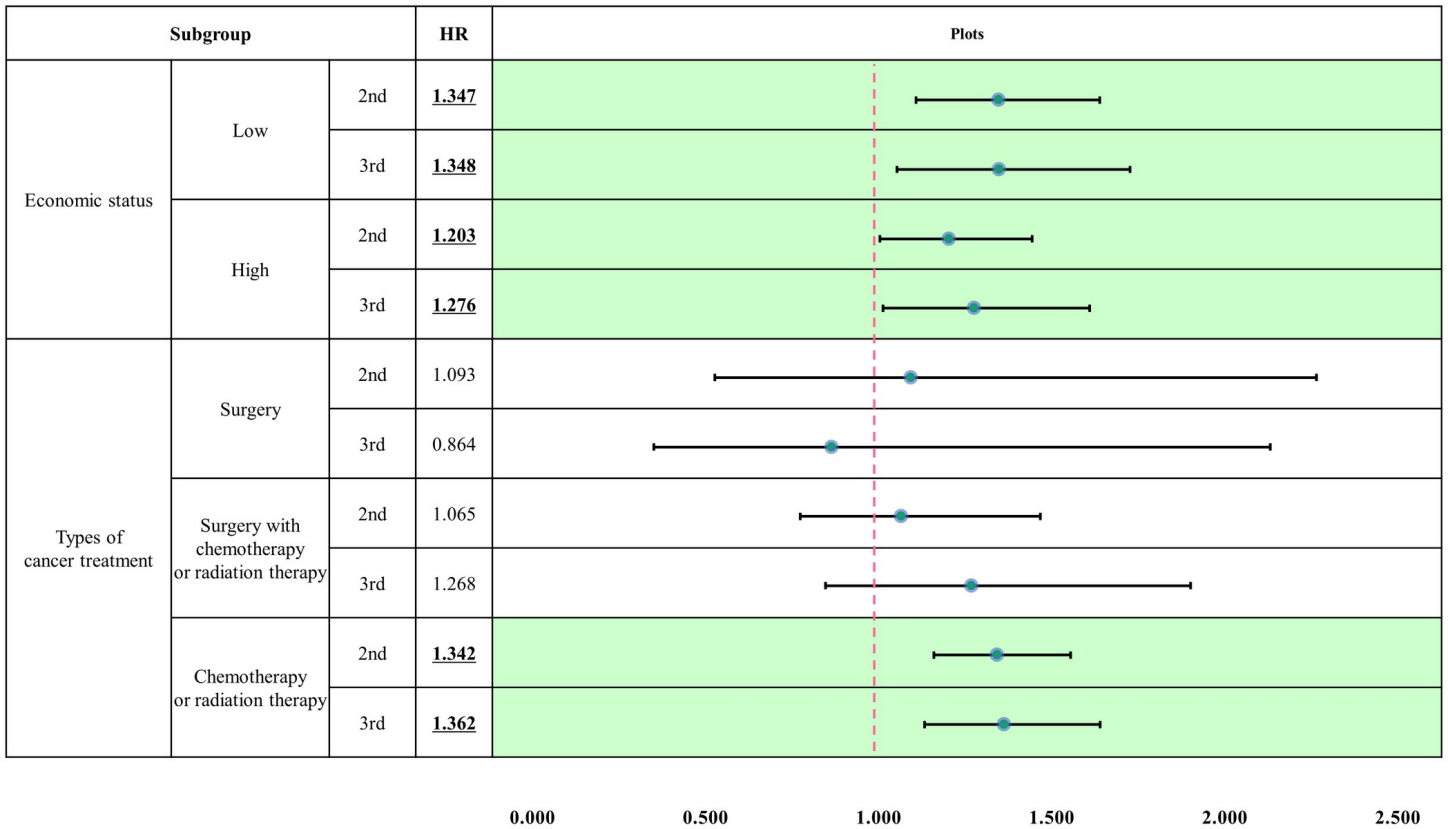
Subgroup analyses for Cox regression per economic status and Charlson Comorbidity Index scores. † This model was adjusted for other covariates, and the reference group comprised patients who visited grade-1 hospitals. 2nd and 3rd denote the nursing grade, and each nursing grade compared to the first nursing grade refers to the effect on the mortality of older adult cancer patients. Plots refers to the HR and 95% CI, which are statistically significant when not including 1.

Fig 3 presents the additional analyses for survival analysis to compare the differences in the association between nursing grade and mortality, according to economic status or type of treatment. In the subgroup analysis, for the low-income group, nursing grade exhibited a higher association with mortality. Regarding the type of treatment, mortality was higher at inferior nursing grades for patients with chemotherapy or radiation therapy, without surgery.

## Discussion

This study revealed that the nursing grades of the initial-treatment hospitals were associated with the positive outcomes of older adult patients with lung cancer. The one- and five-year mortality rates increased at lower nursing grades, which suggests that insufficient staffing precipitates higher mortality in this population. Additionally, a difference was observed in nurse grade’s effect on patient outcomes, depending on the patient’s treatment type and income level. In both low-income and high-income patients, the lower the nurse grade, the higher the mortality rate; however, this effect was greater for low-income patients. In subgroup analysis according to treatment type, the lower the nursing grade, the higher the mortality rate in patients who underwent chemotherapy or radiation therapy.

These results are consistent with previous studies showing shorter length of stays and lower mortality for lung cancer surgery patients in hospitals with nurse staffing [26]. An increase in nurse staffing level has also been associated with a reduced risk of in-hospital death among older patients with hip fractures [27], while a lower nurse-to-patient ratio has been associated with increased patient adverse events [28]. It has also been suggested that an increase in nurse-to-patient ratio is associated with a decrease in rescue failures for patients with cancer, suggesting a volume–outcome relationship [29]. For colon cancer patients, when the nursing grade was lower than that of the initial treatment hospital due to transfer to the hospital, the five-year mortality increased by 1.6 times [30]. This was greater than the effect of nursing grade on mortality in the results of this study, but direct comparison is difficult considering that hospital transfer and changes in nursing grade are mixed.

Considering research findings to date, appropriate nurse staffing during hospitalization is associated with the long and short-term survival of patients. The physical and psychological condition of older adult patients with cancer must be evaluated at the time of initial hospitalization while considering the possibility that other physical problems experienced during the aging process may emerge [7]. Therefore, a multidisciplinary approach is important to maintain the quality of care for older adult patients with cancer [31], and nurses serve as a bridge between medical staff and patients by identifying patient vulnerabilities, providing appropriate interventions, and educating patients throughout the treatment process [7, 32]. Our findings suggest the importance of maintaining a high nursing grade, especially in the treatment of older adult patients with cancer.

From the subgroup analysis, nursing grade’s effect on mortality was greater for low-income patients. Generally, socioeconomic level affects health status—the lower the income level, the more vulnerable individuals are to disease. Previous studies have demonstrated that lower-income individuals are more likely to be diagnosed with terminal cancer and exhibit poorer outcomes [33, 34]. This highlights the need for appropriate treatment and management at initial hospitalization, especially for low-income patients. Insufficient nurse staffing may lead to inadequate nursing care or assessment of patient conditions, which may negatively impact low-income patients and increase their mortality [35, 36].

Depending on the type of cancer treatment, the lower the nursing grade, the higher the mortality rate in patients who have undergone radiation or chemotherapy. The type of treatment for patients with lung cancer is related to the disease progression. Further, lung cancer is rarely detected early, which means that chemotherapy and radiation therapy are used more frequently than surgery [37]. As patients with advanced disease usually receive chemotherapy or radiation therapy, their severity may be relatively higher. Older adult patients with advanced lung cancer may experience various physical and mental changes, including comorbidities and complications. Receiving appropriate treatment at a hospital with a high nursing grade may improve patient outcomes. Appropriate initial treatment for older adult patients with cancer may be crucial to patient outcomes. Specifically, adequate hospital staffing will enable early diagnosis of various disease patterns in patients with early-stage cancer. Therefore, the quality of cancer treatment can be maintained by securing optimal nurse staffing.

This study used representative data and included a large sample of older adult patients with lung cancer; hence, the results of this study can prove valuable in policymaking related to nurse placement. Specifically, the present study can provide policymakers with valuable insights to comprehend nurse staffing’s impact on older adult patients with cancer, and highlight the need for appropriate nurse-to-patient ratios to maintain the quality of cancer care. Additionally, this study provides evidence for the need for appropriate management by underscoring the vulnerability of patients to hospital nursing grade.

However, this study could not determine the stage of patients’ cancer owing to data limitations and health care providers’ detailed information (e.g., specialization such as oncology nurse) was not accessible. Therefore, further studies that consider such information are required. Further, the clinical characteristics of patients not measured in this study may influence their outcomes. We adjusted the clinical severity and treatment method to minimize these effects; however, additional clinical characteristics of the patient should be considered in future studies. Finally, this study only included older adults with lung cancer; hence, additional research on the effect of nursing grade on patient outcomes according to various types of cancer is required.

## Conclusions

As older adult patients with lung cancer exhibit high disease severity and may be physically vulnerable with aging, appropriate treatment in early hospitalization may have a positive effect on patient outcomes. This study reveals that lower nursing grades in initial-treatment hospitals are associated with increased patient mortality. Further, this effect may vary depending on patient characteristics. Therefore, securing sufficient nursing staff is vital for improving the quality of cancer treatment in older adult patients with cancer, and better patient outcomes can be expected in hospitals with higher nursing grades.
